# Increasing the Clinical Potential and Applications of Anti-HIV Antibodies

**DOI:** 10.3389/fimmu.2017.01655

**Published:** 2017-11-28

**Authors:** Casey K. Hua, Margaret E. Ackerman

**Affiliations:** ^1^Department of Microbiology and Immunology, Geisel School of Medicine, Lebanon, NH, United States; ^2^Thayer School of Engineering, Dartmouth College, Hanover, NH, United States

**Keywords:** HIV antibodies, virus neutralization, passive immunotherapy, antibody prophylaxis, antibody engineering

## Abstract

Preclinical and early human clinical studies of broadly neutralizing antibodies (bNAbs) to prevent and treat HIV infection support the clinical utility and potential of bNAbs for prevention, postexposure prophylaxis, and treatment of acute and chronic infection. Observed and potential limitations of bNAbs from these recent studies include the selection of resistant viral populations, immunogenicity resulting in the development of antidrug (Ab) responses, and the potentially toxic elimination of reservoir cells in regeneration-limited tissues. Here, we review opportunities to improve the clinical utility of HIV Abs to address these challenges and further accomplish functional targets for anti-HIV Ab therapy at various stages of exposure/infection. Before exposure, bNAbs’ ability to serve as prophylaxis by neutralization may be improved by increasing serum half-life to necessitate less frequent administration, delivering genes for durable *in vivo* expression, and targeting bNAbs to sites of exposure. After exposure and/or in the setting of acute infection, bNAb use to prevent/reduce viral reservoir establishment and spread may be enhanced by increasing the potency with which autologous adaptive immune responses are stimulated, clearing acutely infected cells, and preventing cell–cell transmission of virus. In the setting of chronic infection, bNAbs may better mediate viral remission or “cure” in combination with antiretroviral therapy and/or latency reversing agents, by targeting additional markers of tissue reservoirs or infected cell types, or by serving as targeting moieties in engineered cell therapy. While the clinical use of HIV Abs has never been closer, remaining studies to precisely define, model, and understand the complex roles and dynamics of HIV Abs and viral evolution in the context of the human immune system and anatomical compartmentalization will be critical to both optimize their clinical use in combination with existing agents and define further strategies with which to enhance their clinical safety and efficacy.

## Introduction

Antibody (Ab)-based therapies have a robust history of therapeutic utility in the setting of infectious diseases, first serving as serum therapy in the 1800s to treat diphtheria and most recently, as monoclonal antibody (mAb) preparations developed to combat emergent outbreaks such as Ebola. Endogenous antibodies raised within the context of HIV infection have similarly demonstrated antiviral activity (1), but typically arise too late in the natural history of infection to prevent disease progression (2). Within infected individuals, viral populations consistently outpace host immune responses in a coevolutionary race to gain functionally favorable mutations contributing to immune evasion or viral neutralization/suppression, respectively. However, heterologous administration of particularly potent and broad antibodies prior to exposure or to acutely infected individuals has demonstrated therapeutic utility in humanized mice (3–7), macaques (8–13), and humans (14–19).

Several reviews have described the activity and potential of broadly neutralizing antibodies (bNAbs) for HIV prevention and therapy (20–27). Building upon a recent comprehensive review of engineering opportunities to extend the functional capacity and antiviral activity of bNAbs (28), this review incorporates findings from more recently published macaque and human bNAb clinical trials to explore both observed and potential challenges to successful bNAb implementation at various stages of exposure/disease to prevent infection, minimize viral spread, suppress viral growth, and eliminate viral populations.

### Promise/Potential: bNAbs in Human Clinical and Macaque Preclinical Trials

**Figure 1:**
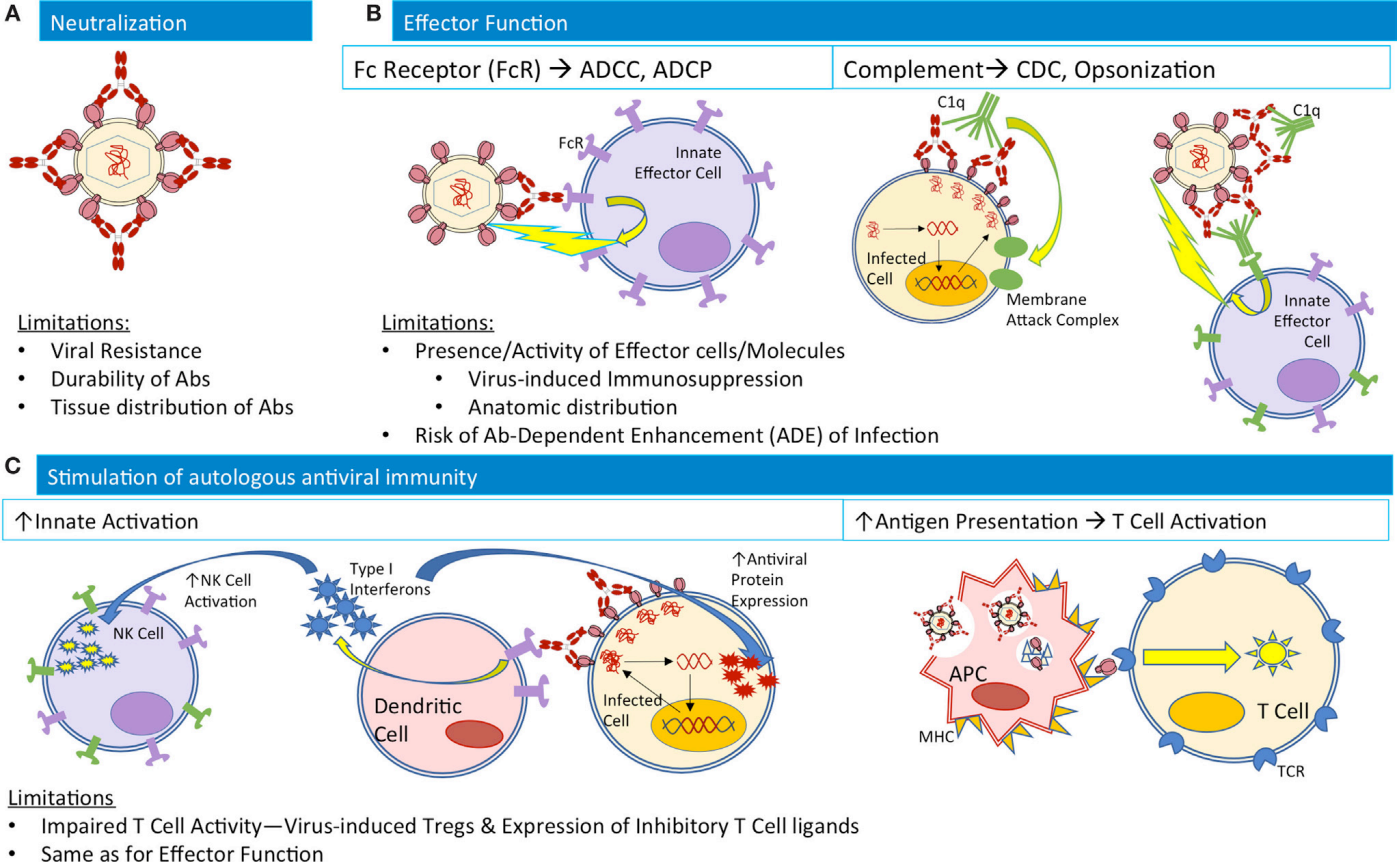
Native Ab functions contributing to antiviral activity and their limitations in the natural course of infection. **(A)** For neutralization, Abs (red) bind viral envelope proteins to block interactions with cellular receptors. **(B)** For effector functions, Abs bound to both viruses and infected cells may engage innate effector cells (purple) to mediate ADCC or ADCP, or complement component C1q (green) to mediate CDC or interactions with complement receptors on innate effector cells for opsonization-based phagocytosis. **(C)** To stimulate autologous antiviral immunity, Ab-bound infected cells may interact with dendritic cells to release type I interferons (blue stars) to stimulate NK cell activation and expression of antiviral proteins within infected cells. Alternatively, antigen presenting cells may phagocytose Ab-virus immune complexes and process viral antigens for presentation to T-cells to mediate cellular immune responses. Abbreviations: ADCC, Ab-dependent cellular cytotoxicity; ADCP, Ab-dependence cellular phagocytosis; CDC, complement dependent cytotoxicity; FcR, Fc receptor; MHC, major histocompatibility complex; TCR, T-cell receptor.

The abundance of studies supporting the antiviral activity and potential of bNAbs to mediate protection from and control of HIV infection in animal models have renewed hope and interest in bNAbs for clinical use. Antibodies can exert antiviral activity through a combination of (1) virus neutralization, preventing initial infection, and viral spread, (2) Fc-mediated effector functions, contributing to the clearance of infected cells, and (3) enhancement of endogenous host antiviral immune responses (Figure 1). In the last 2 years alone, promising human clinical studies to investigate therapeutic benefit in postinfection settings (14–19) and additional preclinical studies to investigate protective efficacy in preexposure/infection settings (29, 30) have clarified the mechanisms of action and efficacy of bNAb administration.

Human clinical studies of VRC01 (14, 17, 31), 3BNC117 (15, 18, 19, 32), and 10-1074 (16) have demonstrated the antiviral activity of bNAbs, offering therapeutic utility in both acute and chronic infection settings. Beyond safety and tolerability, all three bNAbs reduced viral load (15–17) during administration and two, VRC01 and 3BNC117, successfully delayed viral rebound upon discontinuation of antiretroviral therapy (ART) (14, 18). Treatment dosing regimens remain to be optimized and may differ among Abs, dependent upon both the usual considerations of individual mAb pharmacokinetic and pharmacodynamic properties, but also each mAb’s HIV-specific pharmacodynamic properties, such as the slope and completeness of neutralization (33), susceptibility to viral evasion, and propensity to mediate viral (or antigen) trafficking/processing/presentation. In addition, characteristics of individual subjects, such as viral load, diversity, and sensitivity to select bNAb(s) at time of treatment may be considered for more individualized regimens.

Concurrently with direct antiviral activity, treatment with 3BNC117 stimulated and enhanced endogenous antiviral immune responses: in 14/15 viremic individuals treated with 3BNC117, sera from week 24, well after serum levels of 3BNC117 had dropped below detection limits, demonstrated increased breadth and/or potency against a pseudovirus panel as compared to week 0 (19). Interestingly, the increase in neutralization capacity of week 24 sera from ART-treated individuals receiving 3BNC117 was less pronounced than in untreated individuals receiving 3BNC117, suggesting that viral replication and activity contributes to the development of heterologous neutralization (19). Previous studies have also demonstrated the enhancement (13, 34, 35) and importance (36) of autologous humoral and T-cell responses in response to bNAb therapy in macaque models of SHIV [reviewed in Ref. (37)].

The use of HIV Abs in preclinical animal models have similarly demonstrated the potential of mAbs to provide pre- or postexposure prophylaxis, similarly to the early use of immunoglobulins to protect against infection by RSV and Hepatitis A [reviewed in Ref. (38)]. Protection against SHIV acquisition has been demonstrated for multiple bNAbs (9–11, 39–41) with protection dependent upon SHIV strain, bNAb dosage, and bNAb serum concentrations at time of challenge. In models of high-dose SHIV challenge, treatment with ≥5 mg/kg 3BNC117 or 10-1074 successfully blocked SHIV acquisition after a single intrarectal challenge of 1,000 times the 50% tissue culture infectious dose (TCID50), or approximately three times the half-maximal animal infectious dose (42). In a larger study (60 challenged animals vs. 4), the same group determined that serum titers of bNAbs as low as 1:100 were sufficient to prevent SHIV acquisition in ~50% of macaques receiving a single intrarectal challenge at 1,000 TCID50 (8). More recently, the same three bNAbs studied in human clinical trials, VRC01, 3BNC117, and 10-1074, have been tested in preclinical macaque models of repeated low-dose SHIV exposure with impressive results (29). A single infusion of 3BNC117 successfully prevented virus acquisition in models of repeated low-dose intrarectal challenges for up to 23 weekly intrarectal challenges at 10 times the TCID50, whereas control animals acquired infection after two to six challenges. Across the three bNAbs evaluated, the length of protection correlated with Ab potency and half-life. Similarly, in humanized mouse models of HIV acquisition, passive transfer of the bNAb PGT126 demonstrated sterilizing protection against multiple vaginal HIV challenges (30).

### Role of Non-Neutralizing Abs (nnAbs)

As opposed to neutralizing Abs which bind epitopes on functional trimeric Env to prevent cell receptor engagement, nnAbs bind epitopes exposed in non-infective conformations adopted by the unstable Env antigen, such as open Envelope trimers, gp140 monomers, and dissociated gp41 stumps (due to instability or induced by binding to cell receptors). nnAb responses have demonstrated protection through Fc-mediated effector functions and by exerting additional selective pressure and evolutionary constraints upon remaining viruses in humanized mice (43, 44). In a recent study, Horwitz et al. demonstrated the capacity of nnAbs to modulate the course of HIV infection in humanized mice *via* Fc-mediated effector functions in two nnAb cases: (1) using anti-HA Abs in humanized mice challenged with a newly developed recombinant indicator HIV strain containing an HA-tag-, (HIVivoHA) or HIVivoHA-infected cells and (2) using a patient-derived nnAb 246D (45) targeting a linear gp41 epitope in humanized mice challenged with HIV-1_YU2_ virus or HIV-1_YU2_-infected cells (44). In both cases, passive transfer of nnAbs mediated modest protection from viral challenge, reduced viral load in established infection, cleared virus-infected cells, and exerted selective pressure for escape mutations that ultimately deleted or concealed the targeted epitope, all in an Fc-dependent manner that was diminished or absent in passive transfer of the same nnAbs modified with mutations that abrogated binding to activating Fc-receptors (44). Older studies in macaques have suggested that nnAbs may decrease the number of transmitted/founder variants and the viral load in acute viremia, but ultimately did not protect from infection (46–48). Thus while the efficacy of nAbs has been linked to Fc-dependent mechanisms (40) the sufficiency of these antibody activities to drive protection from infection among nnAbs has not been established in NHP. Similarly, the protective capacity of non-neutralizing HIV Abs in humans has been suggested by mother-to-child-transmission studies [reviewed in Ref. (49)] and by the association of V1/V2 nnAbs with protection in the RV144 HIV-1 vaccine trial (50, 51), but remains to be demonstrated.

### Therapeutic Applications and Goals by Stage of Infection

**Figure 2:**
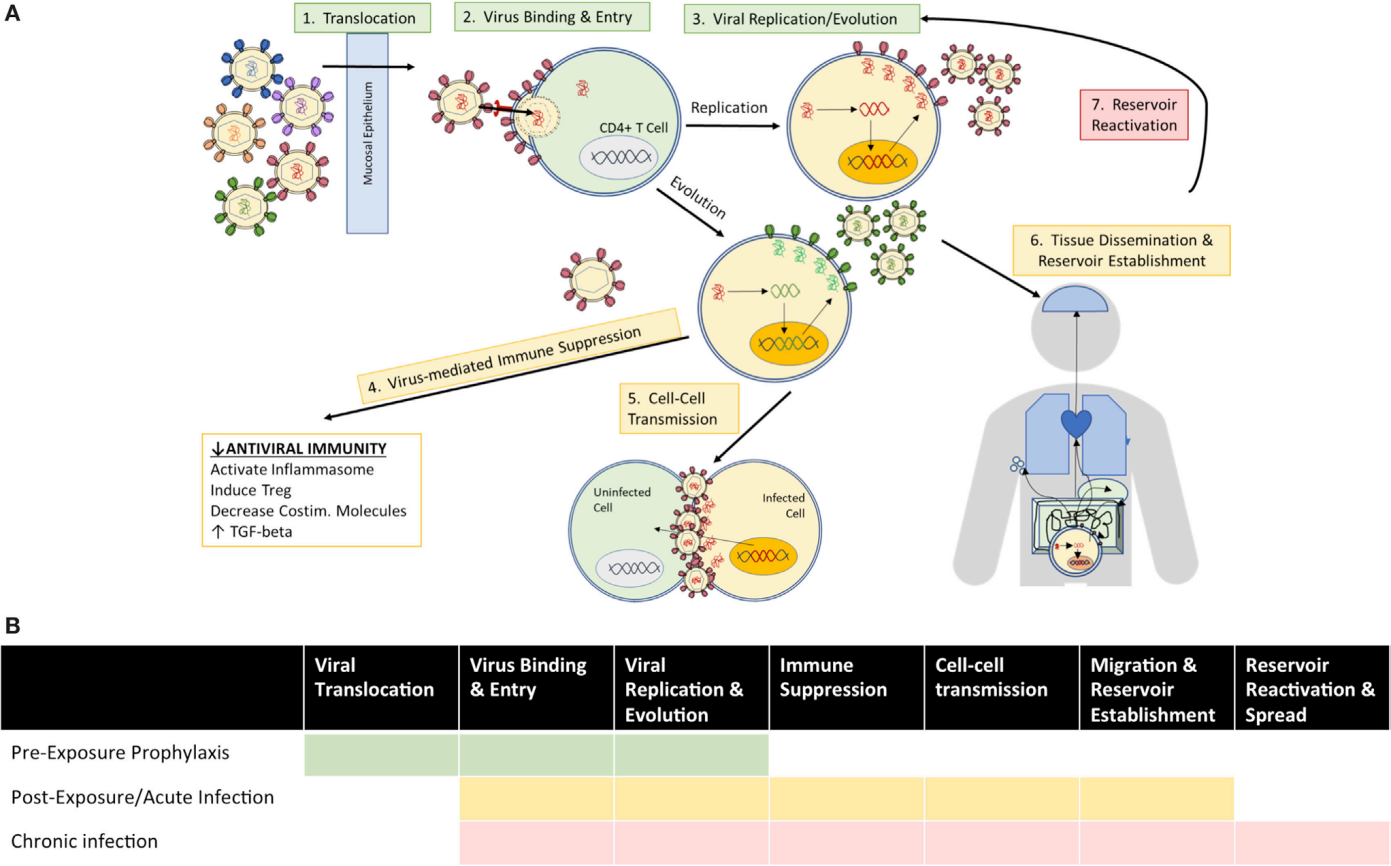
Clinical goals for the use of anti-HIV Abs vary according to **(A)** mechanisms of viral exposure/infection at the time of administration, and **(B)** the viral events which therapeutic Abs seek to inhibit among indicated use prior to exposure (green), as postexposure prophylaxis or treatment of acute infection (yellow), and for treatment of chronic infection (red).

Based on the established roles of mAbs in various infectious diseases, autologous Abs in the natural history of HIV infection, and HIV Abs in clinical and preclinical trials, anti-HIV mAbs find multiple indications for clinical use with therapeutic goals defined by the stage of HIV exposure and disease (Figure 2). Before viral establishment, mAbs could be used either prior to exposure to prevent viral acquisition or postexposure to prevent or limit viral establishment. After viral acquisition in chronic infection settings, therapeutic goals extend to include viral suppression to stabilize and prevent progression of disease, and viral eradication to cure patients entirely of infection. This review investigates the current limitations of and engineering strategies with which to improve the utility of bNAbs at each stage of infection/disease to (1) prevent infection, (2) limit viral establishment/spread, and (3) treat chronic infection *via* suppression of viral growth and reduction/elimination of viral reservoirs (summarized in Table 1).

**Table 1:** Summary table of strategies for the improvement of anti-HIV Ab therapy.

Indication	Goal	Mechanism	Limitation		Improvement Strategies
Vaccine	Block viral entry	Neutralization	Viral resistance	↑ Breadth and potency	Structure-based modifications to ↑ binding
					Broadly neutralizing antibody (bNAb) cocktails
					Bispecific and trispecific bNAbs

			Strict requirement for adherence to dosing schedule	↑ t1/2	FC engineering
					Glycan “masking”
					Carrier proteins, peptides, RBCs

				Continuous Ab expression (adeno-associated virus)	↓ Immunogenicity to ↓ anti-bNAb responses
					Targeting multiple tissues for comprehensive protection
					Enable evolution of delivered Abs: B Cell engineering

			Anatomical distribution	↑ Targeting to sites of exposure	Topical gel delivery
					↑ Binding to mucosal transporters
					Targeted gene delivery

			Risk of Ab-dependent enhancement	↑ Breadth and potency	See above
				Maintain protective concentrations of Abs	Dosing schedule or gene delivery

Postexposure prophylaxis and acute infection	Prevent reservoir establishment	Stimulate autologous antiviral immunity	Insufficient protection after bNAb levels decay	↑ Viral processing and presentation	Coadministration of virus/infected cells (immune complex)
				Counter virus-mediated immunosuppression	Coadministration of immunostimulatory drugs/Abs targeting characterized mechanisms
				Further restrict viral evolutionary space	Identify Abs targeting “non-survivor” epitopes

	Clear acutely infected cells	Ab-mediated Effector functions	Low potency?	Fc engineering for FcR/complement binding	Protein/glycoengineering, subclass switching
				Add toxic payload	Immunotoxin, Ab-drug conjugate

	Prevent cell–cell transmission	Unclear	limited understanding of mechanism	Elucidate mechanism, especially role of Env conformational changes to define “neutralizing” epitopes for cell–cell transmission	

Chronic infection	Suppress viral replication	All of the above (AOTA)	Resistance	Combine with antiretroviral therapy (ART) to suppress replication and opportunities tot evolution	

	Target virat reservoirs	AOTA	Tissue distribution or Abs and reservoir accessibility	Tissue-targeted delivery	Ex: liposomal delivery to central nervous system (CNS)
				Cover diverse populations in compartmentalized tissue	Combine w/additional Abs, ART, latency-reversing agents

			Low Env expression in chronic infection	Target Env epitopes of chronic infection	
				Target non-viral surface markers	All potential reservoir cells, including uninfected (e.g., CD52), or upregulated on infected cells (e.g., CD32a)
				Reactivate reservoirs	Add LRAs

	Long-term clearance of reservoir cells	Autologous T-cell-mediated response	Low cytotoxic T-lymphocyte (CTL) response due to immune suppression	bNAb-based chimeric antigen receptors (CARs)	↑ Clinical safety (↓ risk of CAR mediating infection, synthetic biology “switch” on/off/homing strategies)

			CTL trafficking limitations	Investigate/improve bnAb access to CTL sanctuaries	

	Virol eradication	AOTA	Costs of eliminating reservoir cells in certain tissues (e.g., CNS)	Pair with gene editing strategics so infected cells may survive	

*Overlap of therapeutic goals for listed indications (see Figure 2) are not shown in this table. Goals which are targets for multiple indications are grouped under the indication for which they are the primary focus*.

## Enhancing Preexposure Prophylactic Potential: Preventing Viral Infection

Development of durable protection against HIV has remained a challenge due to the great diversity of HIV species and their adaptive capacity to evade immune-mediated pressure. Viral strains can be described by clade or subtype with viral diversity profiles varying by geographic location, or by neutralization sensitivity designated as very high (tier 1A), above-average (1B), moderate (2), or low (3) sensitivity to Ab-mediated neutralization (pooled plasma samples from four to six clade-matched infected individuals) (52). Clade-matched viral variants are often more sensitive to neutralization by plasma/NAbs from individuals infected by the same clade (52). Thus, the profiling of viral variants endemic to geographical regions could inform the selection of NAbs offering the greatest breadth and potency of neutralization. Ab-based vaccines may function to protect from infection in two ways: (1) neutralization to prevent viral infection in the first place and (2) rapid clearance of virus or virus-infected cells, which will be expanded upon in Section “Enhancing Prophylactic and Therapeutic Potential in Acute Infection: Preventing Viral Reservoir Establishment/Spread.” To offer sterilizing immunity, Abs must offer durable protection with sufficient targeting to anatomic sites of exposure to neutralize viruses and prevent infection. To clear virus and virus-infected cells, Abs must be both readily available at therapeutic concentrations and broadly reactive to maintain efficacy against the diversity of viral strains to which an individual might be exposed. Thus, current and potential limitations to the prophylactic use of bNAbs include: (1) development of viral resistance, (2) requirement for strict regimen adherence, (3) anatomical distribution to sites of exposure, and (4) risk of Ab-dependent enhancement (ADE) of infection.

### Viral Resistance

The arsenal of bNAbs available today targets epitopes spanning a significant portion of the surface of the trimeric HIV Envelope gp140 protein including the V1/V2 loops at the trimer apex, V3 loop glycans, CD4 binding site (CD4bs), gp120-g41 interface, and membrane-proximal external region (MPER) [reviewed in Ref. (53)]. Individual bNAbs vary in neutralization breadth and potency, with some CD4bs targeting bNAbs able to neutralize >90% of global circulating HIV-1 strains at low concentrations (54). However, resistance can develop to even the most potent of bNAbs and has indeed been observed in human clinical trials of all three bNAbs tested thus far (14–17). Even among bNAbs targeting the same epitope, different barriers to resistance development may exist from individual to individual and may arise in part from preexisting bNAb-resistant viral strains. Engineering strategies to combat the development of viral resistance reviewed previously (28) include (1) structure-based modifications to increase the breadth, potency (both neutralization and effector function), and half-life of individual bNAbs, (2) combinations of Abs in cocktail therapies, (3) modifying bNAbs to become bispecific, to carry toxic payloads, or to redirect cells in bNAb-based therapies, and (4) altering delivery strategies.

Since the previous review, three additional studies of newly isolated neutralizing Abs have further supported the importance of structural Ab-Env interactions to neutralization breadth and viral evasion. Demonstrating the importance of Ab binding modes to development of viral resistance, N6, a new bNAb targeting the CD4bs with a novel mode of recognition, does so with amino acid features similar to previously identified mutations to increase the potency of VRC01-class Abs, and demonstrated near-pan neutralization breadth of 98% of HIV isolates tested, including many isolates resistant to other CD4bs antibodies (55). Defining a new neutralizing epitope, the recently isolated/characterized bNAb N123-VRC34.01 recognizes a unique trimer-specific, cleavage-dependent epitope at the N terminus of the gp41 fusion peptide (56). Finally, two recently isolated V2-specific Abs, PGDM1400, and CAP256-VRC26.25, demonstrated unprecedented neutralization potency, protecting against high-dose SHIV challenge at serum Ab concentrations <0.75 μg/mL for CAP256-VRC26.25-LS (57). In addition, these V2-specific bNAbs exhibited neutralization breadth complementary to that of V3-specific bNAb PGT121 against Clade C viruses, ultimately resulting in >90% coverage when used in combination (57).

Recent studies have investigated optimal strategies for combining bNAbs in cocktail therapies (3, 58–61), bispecific formats (62, 63), and novel tri-specific molecules (64). A combination of only three bNAbs targeting different epitopes has been suggested to be sufficient to cover transmitted viral diversity and evolution based on a study conducted in humanized mice (58) and predictive *in silico* models of neutralization breadth and potency (59). In an alternative form of combining epitope specificities, the most potent and broad bispecific Ab to date, 10E8v2.0/iMab, demonstrated 100% neutralization breadth across a 118-member pseudotyped panel with mean inhibitory concentration of 0.002 µg/mL and prevented HIV acquisition in humanized mouse models of infection, demonstrating the synergistic potential of bispecific Abs targeting distinct epitopes (63). In another study, a novel bispecific Ab hinge engineering strategy employing the IgG3 hinge to increase Fab domain flexibility for bivalent binding and to maintain IgG1-Fc function enhanced the *in vivo* therapeutic activity of bispecific bNAbs (62), emphasizing the synergistic avidity-enhancing effect of intratrimeric, heterobivalent crosslinking of Fab arms to increase Ab potency (65). In another novel approach, trispecific Ab molecules containing bNAb specificities against the V1V2 loop trimer apex (PGDM1400), CD4bs (VRC01 and N6), and MPER (10E8v4) were found to mediate increased breadth and potency compared to individual parental bNAbs both *in vitro* and in SHIV challenge models (64). The authors speculated that the tri-specific bNAb may have decreased risk of viral resistance compared to cocktail strategies where differences in component bNAb half-lives may decrease selective pressure (64). However, whether these trimeric molecules engage multiple epitopes simultaneously and/or otherwise confer added benefit over a cocktail consisting of the same three bNAbs remains to be determined.

Beyond development of viral resistance within an individual to bNAb therapy, implications of widespread use of bNAbs as prevention may influence the composition and evolutionary dynamics of worldwide HIV strains. HIV drug resistance is increasingly observed due to poor patient adherence enabling the development of resistance, and subsequent transmission of newly developed drug-resistant strains (66). Similar potential for the development of bNAb-resistant “super-strains” of HIV exists, as bNAb-resistant strains often coexist or arise within individuals from whom bNAbs were isolated. Trade-offs between viral evasion and fitness costs incurred by some resistance mutations (67–70) may mitigate these concerns. However, resistance mutations without fitness costs (70, 71) and the development of compensatory mutations to restore fitness have also been described (67), and antibodies vary with respect to sensitivity to evasion and ease of compensation. Combination strategies such as the cocktails or multispecific molecules described above may best prevent the development of “super-strains” of HIV by further restricting the viral evolutionary landscape. Thus, strategies to optimize bNAb administration and pharmacokinetics to make treatment regimens manageable and supportive of strong treatment adherence will be critical to avoid the development of bNAb-resistance on a more global scale.

### Alleviating Requirements for Regimen Adherence

Because viral rebound quickly occurs upon bNAb decay and renewed replication enables opportunities for viral evolution, protective bNAb dosing schedules must be strictly followed to prevent both viremia and viral resistance. Two methods to decrease the frequency of dosing are (1) increasing the serum half-life of bNAbs and (2) bNAb gene delivery for continuous *in vivo* expression.

#### Increasing Serum Half-Life of bNAbs

Interestingly, bNAb levels decayed more quickly in HIV(+) individuals as compared to controls in human clinical trials, potentially due to the formation of Ab-virus immune complexes in infected individuals that are more rapidly cleared from circulation. For bNAbs to offer prevention potential, and to avoid the development of resistance, serum half-life would need to be long enough to maintain protective concentrations at reasonable dosing schedules. Fc engineering strategies to increase the half-life of bNAbs have been described [reviewed in Ref. (28, 72)], including studies of the VRC01-LS variant which demonstrated a threefold longer serum half-life and increased translocation to mucosal tissues, ultimately leading to improved potency and protection against high-dose rectal challenge in non-human primates (29, 73, 74). VRC01-LS (M428L and N434S) (29, 74) has now advanced into Phase I clinical trials (NCT02797171, NCT02840474, NCT02599896, NCT02256631).

#### Continuous Protection *via* Gene Delivery: *In Vivo* Expression of bNAbs

In an indirect way to extend the lifetime of bNAb therapy, gene delivery has been increasingly explored to achieve durable Ab concentrations, most prominently by adeno-associated virus (AAV) vectors [reviewed in Ref. (75)]. Historically, AAV delivery-based gene therapy has demonstrated safety and efficacy in both macaques (76–79) and humans (80–85) for a variety of diseases, and has become the first clinically and government-approved gene therapy in Europe (86, 87). Within the realm of HIV, AAV-delivered HIV-specific bNAbs and Ab-like molecules such as CD4-Ig have demonstrated sterilizing and durable protection against SIV/SHIV infection in macaques (73, 88–90) and HIV infection in humanized mice (4, 91), and are now undergoing Phase I human clinical trials to evaluate safety, deliverability, and potential efficacy in England (NCT01937455).

Current limitations to bNAb gene delivery include the development of anti-bNAb responses and the virus independence of bNAb expression. First, several studies of AAV-delivered bNAbs to macaques have demonstrated the development of anti-bNAb responses (73, 88, 90, 92), despite “rhesus-ization” of bNAbs and addition of immunosuppressive therapy, potentially due to immune-stimulating effects of the AAV itself which can trigger innate pattern recognition receptors and toll-like receptors or engage preexisting cellular (93) or humoral (94) immunity. Side-by-side comparisons of anti-bNAb responses in passively transferred bNAbs vs. AAV-delivered bNAb treatment have been proposed to delineate immunogenic contributions from AAV vs. Ab (75). Engineering strategies to decrease the immunogenicity of AAV capsids and coadministration of immunosuppressive agents (cyclosporine, T-cell inhibition, IVIG, corticosteroid) have been proposed and shown promise (75). However, immunosuppressive agents may also decrease bNAb Fc-mediated effector function and the development of autologous antiviral responses, placing the bulk of protection on neutralization. Thus, studies to determine the costs and benefits of adding immunosuppressive agents to AAV-delivery regimens are warranted.

Second, current AAV-delivery of bNAbs results in bNAb expression independent of viral trafficking, replication, and evolution, and therefore (1) may not be ideally distributed for prevention of infection/reservoir establishment and (2) cannot respond to changes in the viral population. Intramuscular delivery of vectored gene therapy to skeletal muscle is most extensively studied thanks to muscle tissue’s amenability to long-term gene expression, abundant vascular supply for quick transport to the systemic circulation, and ease of accessibility (95). However, vectored gene delivery to additional tissues including the liver, brain, spinal canal, skin, and eyes have been described (95). Targeted gene delivery to these tissues may be especially useful if protective Ab concentrations in these tissues are not possible from circulation alone.

However, such bNAb-expressing tissues are unable to respond to viral evolution, and may become less useful as viral populations develop resistance to the administered Ab. Thus, strategic delivery of bNAb genes to B-cells for integration at native BCR loci (gene targeting into the *Igh* locus) under the normal regulation of heavy-chain expression, Ab class-switching, and somatic mutation may offer the added benefit of coevolution with viral populations. A similar technology of *in vivo* bNAb-as-BCR evolution has been used in HIV Env immunogen studies in transgenic knock-in mice containing B-cells expressing germline heavy chain variants of VRC01-class Abs (96–98), which were successfully activated/expanded and underwent somatic hypermutation in response to various Env immunogen regimens. Viral challenge of similarly generated knock-in mice containing genes for mature bNAbs as BCRs may demonstrate proof-of-concept for bNAb-based BCR engineering. Clinical translation of such a strategy could parallel chimeric antigen receptor (CAR) T-cell procedures, whereby B-cells could be extracted from a patient and engineered *ex vivo* to expressed bNAb-based BCRs prior to reinfusion. Investigations into efficient and targeted IgH knock-in would be critical to this approach and increased understanding of B-cell differentiation and subtypes, BCR editing, and tolerance checkpoints would be beneficial. Additionally, switchable gene expression may be desired to prevent unchecked expansion/growth. While this ability to coevolve may not ultimately provide any benefit, natural infection histories provide both reasons for optimism and pessimism. In favor of the optimistic possibilities, the ability of bnAbs to improve autologous antibody neutralization potency, and their ability to collaborate with other lineages for beneficial outcomes suggests that the ability to adapt over time could be advantageous.

### Targeting Anatomical Sites of Exposure

One probit analysis of bNAb-treated macaques suggested that a serum level of 100 times the bNAb IC50 affords 50% protection against intrarectal infection (41), a level that is estimated to be attainable by biannual passive Ab injections given the serum Ab levels and half-lives of VRC01 and 3BNC117 in human clinical trials (25). In an SHIV macaque study, IV infusion of 2 mg/kg PGT121 completely protected subjects from intravaginal challenge with 5 × 10^4^ TCID50 SHIV-SF162P3, with no detectable viral RNA or DNA found in distal tissue sites by day 10 after challenge (99). However, concentrating Abs at the sites of viral exposure may allow even lower doses to be protective. Because viral exposure often occurs at mucous membranes including the rectal and vaginal tracts, the presence of bNAbs at mucosal sites to mediate immune exclusion may improve protection. Therapeutic administration and Ab engineering strategies to improve bNAb use for mucosal immunity were described previously (28) and included topical gel delivery, Fc engineering to enhance binding to FcRn and pIgR at mucosal sites, and designing IgA and chimeric IgGA variants of bNAbs. In addition, some of the strategies described above such as targeted AAV-delivery of bNAb genes to specific tissue sites or BCR engineering to express class-switched IgA versions of bNAbs may be beneficial. Studies have found contrasting evidence for (100–103) and against (104) a role for bNAbs, formatted as various isotypes, in preventing transepithelial migration. The reason for this discrepancy is unknown but may be related to the utilization of older-generation or less potent bNAbs in the prior studies (2F5, 2G12, 4E10), whereas the most recent studies investigate newer-generation, more potent bNAbs. In that study of bNAbs targeting a wide range of epitopes, bNAbs did not block the transcytosis of either cell-free or cell-associated HIV-1 *in vitro* and instead relied upon neutralization to decrease the infectivity of transcytosed viruses (105). Thus, increasing the local concentration and neutralization breadth and potency of bNAbs at mucosal sites may enhance protection against mucosal infection.

### Potential Risks: ADE of Infection

Thus far, ADE of HIV infection has only been observed *in vitro* and grouped into complement- (106–108), Fc Receptor (FcR)- (109–112), and conformationally mediated (113, 114) mechanisms which ultimately facilitate virus internalization or receptor-independent virus-cell membrane fusion [reviewed in Ref. (115, 116)]. In addition, antibody-virus immune complexes could increase trafficking of infectious virions to lymph nodes, thereby amplifying rates of viral infection and replication. While debate exists over whether ADE occurs in natural HIV infection, the presence of enhancing Abs have been correlated with disease progression in some studies of sera from HIV-infected individuals (117, 118) [but not others (119)] and suggested to explain increased rates of infection in individuals with relatively low Ab responses in vaccine trials (120) and correlations of particular FcR genotypes characterized by stronger Fc-binding affinities with higher infection risk (121, 122). Both nnAb and neutralizing Ab at subneutralizing concentrations can enhance infection *in vitro* (109), and epitope specificity does not necessarily determine an Ab’s potential for ADE (115). Thus, maintaining protective concentrations of bNAbs *via* repeated dosing or continuous expression (AAV) may be especially critical to decrease the risk of ADE.

## Enhancing Prophylactic and Therapeutic Potential in Acute Infection: Preventing Viral Reservoir Establishment/Spread

After exposure, bNAbs may be used as prophylaxis to prevent the establishment and spread of viral reservoirs [reviewed in Ref. (123, 124)]. Successful elicitation or administration of HIV-specific Abs in macaque models of acute SHIV challenge and infection have correlated with reduced acute viremia and limited reservoir seeding (46, 125, 126). The window for postexposure prophylaxis has been estimated to be as short as 24 h to block infection by cell-free virus in macaque models of SHIV infection (127, 128) and within the first 9–10 days to limit viral reservoir seeding and spread (129). Resistance continues to be a major concern for all of the described bNAb indications in this review, but may be especially relevant in postexposure settings where autologous viral populations may be screened for preexisting resistance to bNAbs. Mucosal barriers and/or autologous immune responses often limit the diversity of transmitted/founder (T/F) strains; in one study, 80% of individuals infected through heterosexual and 60% through homosexual contact were found to have a single founder virus strain (130). Thus, the low viral diversity present in acute postexposure settings render it a particularly useful time at which to screen viral populations to inform the choice of bNAb(s) therapy. Efforts to adequately sample viral diversity later during chronic infection become more difficult as latent reservoirs are established and thus viral sensitivity screening may be less useful at later time points.

In addition to the previously described goals to neutralize virus to prevent initial infection, postexposure prophylactic use of Abs additionally seeks to limit reservoir seeding and spread. Enhancing the ability of mAb therapies to (1) increase autologous immune responses and (2) target acutely infected cells represent two strategies by which to accomplish this goal.

### Increasing Protection by Influencing the Autologous Adaptive Immune Response

Both neutralizing Ab and nnAbs depend upon Fc-mediated effector functions for antiviral activity *in vivo* (131, 132). Through the Fc portion, elicitation of even nnAb responses offers therapeutic utility, demonstrating protective effects in both vaccination (121, 133) and passive transfer studies (44, 134, 135). Stimulation of autologous Ab responses, whether neutralizing or not, thus remains a promising means by which to generate durable effects from Ab therapy.

Broadly neutralizing antibody therapy has been associated with enhanced autologous antiviral immune responses in both human (19) and macaque (13, 34, 35) studies. Proposed mechanisms for this observed effect include (1) facilitation of viral processing and presentation, (2) potential immune-stimulating effects in an otherwise suppressed adaptive immune background conferred by HIV infection, and (3) restriction of viral evolutionary space by both administered bNAbs and elicited autologous Abs. Efforts to increase autologous Ab responses may thus focus upon enhancing each of these mechanisms.

#### Enhancing Viral Processing and Presentation

Increasing the effector function capacity of anti-HIV Abs by Fc engineering to skew binding toward particular Fc receptors represents one mechanism by which to engage and stimulate endogenous immunity, and has been previously reviewed in Ref. (28, 72). Beyond engineering bNAb molecules, adjunctive coadministration of envelope, virus or infected cells with Abs in immune complexes can engage FcɣRs on antigen-presenting cells to facilitate antigen internalization and enhance APC activation and presentation, ultimately “boosting” endogenous antiviral immunity [reviewed in Ref. (136)]. Although one study found that opsonization of HIV-1 with polyclonal anti-HIV IgGs was associated with decreased dendritic cell activity (137), further investigations of Abs of varying neutralization potency formatted as different isotypes have been proposed to clarify the generalizability of that study (136). In another study, administration of HIV-1 gp120 Env and a CD4bs mAb resulted in enhanced neutralization potency of elicited humoral responses in mice (138). Notably, Fab-mediated effects that resulted in greater presentation of particular epitopes in the Ab-bound immune complex were determined to be at least partially responsible for the increased neutralization potency of the elicited antibody response (139–141). Additional parameters to be investigated in the use of immune complexes to stimulate endogenous antiviral immunity include antigen format (soluble vs. virus vs. infected cell), Ab format (neutralization capacity, isotype, Fc variants), ideal ratios of Ab:Ag to form complexes, and routes of administration (136).

#### Combatting Viral-Mediated Suppression of the Antiviral Immune Response during Acute HIV Infection

Acute HIV infection is characterized by early suppression of antiviral immune responses to support viral growth and spread. Mechanisms for this antiviral-specific immunosuppression include increased activation of the NLRX1 inflammasome (129, 142), which negatively regulates interferon-stimulated antiviral genes, and increased secretion of TGF-beta (129) that inhibits adaptive immune responses. In addition, viral interactions can induce early activation of regulatory T-cells (143, 144), and increase the expression of inhibitory T-cell markers PD-1 and CTLA-4 (145, 146). The effect of these immunosuppressive mechanisms on Ab-mediated effector function remains to be determined (124), but likely decreases the efficiency with which Ab-mediated stimulation of autologous immune responses arise. Combination with immunostimulatory drugs and antibodies targeting these specific mechanisms of immunosuppression may thereby increase the development of autologous antiviral immune responses, but may be a double-edged sword as there is a concomitantly increased risk of enhancing the development of anti-bNAb responses or the pool of CD4+ T-cells available for infection. *In vivo* studies of such approaches will be especially critical to determine the utility and/or feasibility of this approach.

#### Identifying Abs Targeting “Non-Survivor” Epitopes: Limiting Viral Evolution

Finally, autologous Ab responses may have antiviral effects by limiting the space for viral evolution through the targeting of “non-survivor” epitopes, regions in which resistance mutations incur survival costs or complete lethality [reviewed in Ref. (43)]. These epitopes can be distinct from epitopes recognized by bNAbs, against which resistance mutations commonly develop and are often contemporaneous with the presence of the bNAb in individuals from which they are isolated. Thus neutralizing epitopes identified thus far are largely “survivor” epitopes and a recent review has raised the concern of “survivor bias” in present studies of protective humoral responses (43). Potential non-survivor epitopes include functionally critical regions targeted by non-neutralizing epitopes that become exposed upon conformational changes including CD4-inducible epitopes (147) and gp41 epitopes like the fusion peptide (46, 56): passive transfer of nnAbs targeting these regions successfully decreased the number of transmitted/founder viruses from high-dose SHIV challenge in macaques (46).

### Clearing Acutely Infected Cells

Acutely infected cells must be cleared early to prevent the establishment of reservoirs. Toward this goal, Abs can engage innate effector cells through the Fc portion to stimulate Ab-dependent cellular cytotoxicity (ADCC), Ab-dependent cellular phagocytosis (ADCP), or complement-dependent cytotoxicity (CDC). To further improve Abs’ capacity for cell-clearance, bNAbs may be engineered for enhanced Fc-mediated effector functions [described previously in Ref. (28, 72)] or modified through the conjugation of toxic payloads [reviewed in Ref. (148)].

#### Enhancing Ab Effector Function

Engineering strategies to augment Fc-mediated effector functions of HIV Abs were described in detail previously (28, 72), including IgG subclass switching and protein/glycoengineering to bias Fc receptor/complement component binding profiles. Multiple Fc-engineered mAbs have now entered and/or demonstrated safety and efficacy in various phases of clinical trials as well [reviewed in Ref. (149)]. The results of these studies will inform the capacity of *in vitro* and animal models of Fc-engineered Ab function to predict effector function in humans. They may further help to model the relationships between changes in Ab-Ag binding affinity, Fc-Fc receptor binding affinities, and clinically significant differences in effector functions in humans as has been described in animal models (150–152), and to determine whether there is an optimal Fc receptor binding affinity profile to elicit particular effector functions.

#### Immunotoxins

In acute infection, potent, transient cytotoxicity may be sufficient to inhibit reservoir establishment. Thus, conjugation of Abs with more toxic payloads such as bacterial exotoxins may be tolerable as a short-term solution to ensure rapid and complete cytotoxicity in place of or in addition to Fc-mediated effector functions to treat acute infection. In contrast, Ab-based immunotherapies that are more amenable to long-term use with more durable effects will be discussed in Section “Enhancing Therapeutic Potential for Chronic Infection” to treat chronic infection. In addition, viral Env has been suggested to be more highly expressed during early infection (153), making viral Env-targeting Abs potentially more useful as targeting agents during this period.

In one study, HIV-specific recombinant immunotoxin (RIT) employing Pseudomonas exotoxin A, 3B3-PE38, in combination with ART significantly decreased the number of HIV RNA-producing cells compared to ART alone in BLT humanized mouse models of HIV infection (154), although a potential for toxin immunogenicity and viral resistance were cited as limitations to chronic use of the immunotoxin. In a recent study testing a panel of HIV-specific mAbs as RITs, epitope specificity was found to correlate most with cytotoxicity against H9/NL4-2 cells (HIV Env expressing cell line), as compared to binding/neutralization potency (155). The most effective RIT employed mAbs targeting a non-neutralizing epitope in the gp41 loop region, which lies close to the plasma membrane and may thus allow the toxin to enter the cell more effectively (155, 156). Combination with soluble CD4 (sCD4) further increased the cytotoxicity of gp41 loop-targeting RITs, likely due to increased exposure of the gp41 epitope after sCD4 binding induced conformational changes in Env and increased internalization of Env-bound RITs in the presence of sCD4 (155, 157).

*In vivo* studies of another gp41-specific RIT employing a Ricin A chain (RAC) toxin, 7B2-RAC, also demonstrated efficacy in SHIV-infected macaques prior to the development of antidrug Abs after 2–3 weeks due to RIT immunogenicity (158). In the same study, to combat this observed immunogenicity, the authors PEGylated RITs prior to use in mouse models of HIV, which resulted in lower antidrug Ab levels in a subset of mice (158). However, additional methods to decrease RIT immunogenicity [reviewed in Ref. (159)] may be required. In addition, cytotoxic payloads with decreased immunogenicity may be used instead of protein toxins to make antibody-drug conjugates (ADCs). In the SHIV macaque study of 7B2-RAC, ADCs employing existing small molecule cytotoxic drugs were also tested but were less efficacious than the RIT, likely because their drug toxicities were 1-log less potent than the RAC toxin (158). Thus, ADCs may become more competitive as more potent cytotoxic small molecule drugs are developed to rival recombinant toxins.

### Preventing Cell–Cell Transmission

In addition to infection by free HIV, cells may become infected by horizontal transmission from other infected cells [reviewed in Ref. (160)]. The frequency with which cell–cell transmission occurs *in vivo* is unknown, but infection by cell-associated virus has been demonstrated in Macaque models of infection by SHIV-infected splenocytes (161), and suggested by studies of mother-to-child transmission of HIV during pregnancy, labor, and delivery [reviewed in Ref. (162)] and by spatial segregation of viral sequences (163). In addition, cell–cell transmission of virus was found to be more efficient than infection by free virus *in vitro* (164) and could lead to multiple infections of a single cell (165). A recent study found that different bNAbs exhibited Ab- and viral strain-dependent capacities to inhibit cell–cell transmission: for non-CD4bs-epitope targeting Abs, mAbs with increased potency of free virus neutralization exhibited greater losses in neutralization activity of cell–cell transmission, suggesting that optimal binding characteristics for free virus neutralization differ from those for cell–cell transmission neutralization (166). In another recent macaque study, bNAb PGT121 administered at protective concentrations against cell-free virus were only partially efficacious (3/6 macaques) at protecting from SHIV-infected splenocyte challenge (161). Studies to elucidate the mechanisms by which cell–cell transmission occurs and conformational differences in Env structure during transmission (167) would be beneficial to defining a strategy to improve this type of neutralization.

## Enhancing Therapeutic Potential for Chronic Infection

Current therapy for chronic infection aims to suppress viremia to prevent symptoms from virus-stimulated immune activation and to prevent the growth/spread of viral reservoirs to preserve CD4+ T-cells. Today, ART largely accomplishes these goals to maintain low viral loads by blocking viral replication, but its use is limited by long-term end-organ drug toxicities, a strict requirement for treatment regimen adherence, and the development of viral resistance (168). In addition, persistent low-level viremia can remain even under ART treatment (169–171), potentially from cells infected prior to therapy initiation or in tissues with poor drug penetration (172) or residual virus replication in latently infected cells (169, 173, 174). Thus, therapeutic alternatives for chronic HIV infection that may lessen the burden or address limitations of ART are desired.

Encouraging results for the utility of bNAbs as treatment for chronic infection [reviewed in Ref. (26, 168)] from recent human clinical trials include effective suppression of circulating free virus in individuals harboring bNAb-sensitive strains (15, 17), delayed viral rebound after ART treatment interruption (14, 19) suggesting reduction of cell-associated virus or viral reservoir size (32), elicitation of host immune responses (19), and suppression of HIV replication in reservoir cells (175). Most of these results were found in a subset of treated individuals, dependent upon the preexisting resistance of circulating/reservoir strains, and in all cases viremia rapidly rebounded upon bNAb decay or cessation. Thus, strategies to combat both preexisting and *de novo* development of viral resistance remain a target of Ab therapy for chronic infection.

### Combination with ARTs

Given the relative success of existing ART in treating chronic HIV infection, the comparison between bNAb therapy vs. ART or the benefit of adding bNAb therapy to ART has garnered interest. The potential for bNAbs to enhance the effects of ART lies in the ability to address residual sources of viral replication and further limit the development of viral resistance. One study found that the combination of bNAbs with ART was no better than treatment with ART alone in macaque models of SHIV infection (126), likely due to the already low level of viral replication and in some cases undetectable viremia of subjects undergoing ART alone in the observed period. On the other hand, ART significantly limits, but may not completely prevent, viral evolution of both circulating and tissue reservoir populations (176, 177). Thus bNAbs may be especially useful in combination with ART, which removes the major limitation of evolving resistance. In addition, the tissue distribution of ART and bNAbs or bNAb-based therapies may complement each other, with bNAbs “cleaning up” after persistent viral replication from virus-infected cells in tissue compartments receiving subtherapeutic levels of ART, such as lymph node germinal centers which may be more readily accessible to Ab- or Ab-based bispecific molecules interacting with APCs or T-cells (168). On the other hand, ART-mediated suppression of viral replication decreases the expression of Env epitopes on the surface of infected cells, and may thereby require more potent bNAbs or Abs targeting non-Env markers of infection.

### Targeting Viral Reservoirs: Accessing Tissues and Identifying Cell Targets

Distinguishing which tissues and cell types can support viral reactivation and/or contribute to AIDS progression is critical to defining the extent of viral eradication desired/needed and the development of strategies with which to target cellular reservoirs. For viral remission, accepting persistent viral latency in some reservoirs with low reactivation potential and/or high costs of cellular/tissue damage may be acceptable. Multiple studies have suggested that decreasing the size of the viral reservoir delays viral rebound after ART is stopped (178–180), with one modeling study suggesting that a four-log reduction of the simulated 3 × 10^5^ member reservoir size comparable to observed reservoirs of 10^5^–10^7^ (181) could prevent viral rebound after ART altogether (182).

#### Tissue Reservoirs: Distribution and Accessibility

Viral reservoirs may establish in multiple tissue sites (183) and cell types (184), making sufficient access to and efficacy in reservoir tissue sites and identification of target cells key components of combatting latent HIV infection. The primary site for viral replication occurs in central lymphoid tissues (18, 19), with lymph nodes, spleen, and GI tract lymphoid tissue harboring the largest numbers of HIV-infected cells (183). Unfortunately, these secondary lymphoid organs can act as pharmacologic sanctuaries limiting ART concentrations and viral suppression: lower concentrations of ART in lymph nodes (vs. blood) have been associated with persistent viral replication within lymph nodes (185). However, viral RNA/DNA has been found in nearly all tissues, including immune-privileged sites such as the central nervous system (CNS), testes, and placenta (183). Mixed evidence for compartmentalization, or differences in viral populations among different tissues and in circulation, exists (183) and may indicate a need for combination therapy with additional Abs, ART, or latency reversing agents (LRAs) with wider tissue penetration or more tissue-specific administration/targeting, such as liposomal delivery of drugs to the CNS [reviewed in Ref. (186)].

#### Reservoir Cell Types: Surface Markers of Infection

Within individual tissues, CD4+ T-cells comprise the majority of cell types harboring latent virus but viral DNA has been found in non-CD4+ T-cells [reviewed in Ref. (187)], including CD4−/CD8− T-cells (188), macrophages [reviewed in Ref. (189)], monocytes, tissue macrophages (190), and follicular dendritic cells (191, 192). Identifying reservoir cells can be challenging due to their relative quiescence and transient expression of low levels of viral antigens. Expression of HIV Env may additionally be different in latent cells as compared to cells with active viral replication. Given the instability of trimeric Env, non-neutralizing epitopes accessible on monomeric gp140 or gp41 stumps have been suggested to be displayed on the surface of infected cells over time (193). Thus, epitope targets of therapeutic HIV mAbs for chronic infection may vary significantly from those for the acute postexposure setting, reflective of the differing goals of targeting latent cells vs. active virus.

One strategy to combat this challenge is to identify non-viral surface markers that are expressed, or preferably upregulated, on infected cells. In an extreme example, CD52 expression on a wide breadth of immune cells capable of serving as reservoirs during HIV infection—nearly all T-cells, B-cells, and plasmacytoid dendritic cells—may be targeted by anti-CD52 Abs to deplete reservoir cells (194, 195), but uninfected immune cells may also be affected. Instead, Abs recognizing markers suggested to be upregulated by infection (196) may preferentially target reservoir cells and ameliorate some of the side effects expected from more general immune depletion strategies. In addition, these Abs may be used to guide the delivery of more toxic payloads in Ab-based therapies such as immunotoxins, bispecific T-cell engagers, or CARs in cellular therapy.

In another approach, LRAs may be used to re-activate cells and increase expression of viral antigens. However, the reactivation of virus increases the production of viral particles and risk of increasing cellular infection rates, and therefore must be balanced with potent elimination therapy, including bNAbs, in “shock and kill” strategies to quickly and efficiently eliminate reactivated cells. Coadministration of bNAbs with three viral inducers in humanized mice reduced the proportion of mice with viral rebound after Ab levels decayed, whereas Abs alone or combinations of bNAbs with a single inducer failed to affect viral rebound rates (132). Thus, strategies to optimize the combinations of Abs and inducers (25, 197) or to increase the potency or long-term effects (e.g., autologous immune responses) of Abs as elimination therapy may be necessary to maintain viral suppression after the decay of therapeutic Ab.

### Long-term Clearance of Infected Reservoir Cells: Cellular Therapy

Natural Abs rely upon Fc-mediated effector function to clear infected cells. However, Ab-mediated effector functions may be less active or unavailable in infected tissue reservoirs with immunosuppressed or immune-privileged microenvironments. Thus, an alternative strategy to increase the potency with which Abs may destroy infected cells focuses upon addressing the limitations of T-cell-mediated responses. Effective cytotoxic T-cell responses have been associated with viral control in studies of relatively rare long-term non-progressors (198, 199) and HIV-exposed seronegative individuals (200). Similarly, persistent viral suppression after Ab therapy in a subset of SHIV-infected macaques (3 out of 18) was associated with improved host virus-specific cytotoxic T-lymphocyte (CTL) responses (13). Thus, anti-HIV Abs may be used to augment or complement cellular immune responses for long-term term viral control.

#### Engineering for Enhanced Cytotoxic Responses: CAR Cells

Rather than relying upon the natural development of host CTL responses, an alternative strategy employs HIV-specific Abs to re-direct T-cells toward HIV-infected cells. Promising bispecific T-cell engaging molecules (201, 202) and CAR T-cells (203, 204) have been previously reviewed (28) and are increasingly viable given the recent advent of the FDA’s first recommendation for clinical approval of a CAR T-cell therapy (Novartis CTL019). Strategies with which to enhance the cytotoxic activity of bispecific T-cell engaging molecules and HIV-specific CAR T-cell approaches were described previously (28). This review thus focuses upon strategies with which to improve the clinical safety and efficacy of CAR therapies for HIV infection.

One concern is that HIV-binding CARs may render T-cells more susceptible to infection, especially CD4ζ-based CARs (205). Thus, strategies to protect anti-HIV CAR-modified cells include the cotransduction/expression of fusion inhibitors (206, 207), and knock-out/knock-down of CCR5 expression (208–211). A second concern is that the necessary expansion of engineered T-cells can lead to exhaustion and loss of activity (205), compounded by the fact that T-cells often already express inhibitory markers associated with exhaustion during chronic HIV infection (145, 146). To combat this predisposition for T-cell exhaustion, stem/progenitor cells may be modified with CARs instead with the added benefits of the generation of more durable and potentially diversified cell types bearing the CAR, as well as the built-in thymic immune tolerance checkpoints through which T-cells developing from stem/progenitor cells must proceed (205). Hematopoietic stem/progenitor cells modified with a CD4ζ-CAR in humanized mouse models of HIV infection successfully differentiated and maintained CAR expression in multiple cell types, including T-cells and NK-cells, and reduced viral loads in treated animals (204).

More general concerns with the clinical use of cellular therapies as a class have been reviewed (212), and include the potential for cytokine storm from mass T-cell activation and cytotoxicity (213, 214), cellular transformation from genomic integration of viral vectors due to insertional mutagenesis (215), and autoreactivity (216). Strategies to mitigate these risks employ synthetic biology tools [reviewed in Ref. (217)] such as inducible suicide or “switch” strategies to induce apoptosis of CAR T-cells (218, 219), feedback-based “pause” switches (220), and preferential homing/activation based on “logic gate” requirements for engagement of multiple antigens (221–225).

#### Complementing Autologous T-Cell Responses: Access to T-Cell Sanctuaries

Cytotoxic T-lymphocyte trafficking patterns may limit their ability to access all viral reservoir sites (226). In one macaque study of SIV infection, the viral reservoir population of elite controllers was found to differ from that of progressors: elite controller macaques largely harbored virus in follicular helper T-cells (T_FH_) whereas progressor monkeys harbored virus across a wider breadth of T-cell subtypes (226), suggesting that protective CTL responses may not be able to access T_FH_ reservoir cells. Thus the ability of bNAbs (or other anti-HIV Abs) to access and clear reservoir cells from CTL sanctuaries (such as T_FH_s in B-cell follicles) is of particular interest (25).

### Potential for a True “Cure”: Viral Eradication vs. Reservoir Eradication

A true HIV “cure” would entail the complete eradication of virus from an infected individual, including all latent reservoir cells. By this definition, an extremely potent form of “shock-and-kill” strategies would likely be necessary to expose and eliminate all reservoir cells using HIV mAbs. In addition, the tangled link between viral eradication and tissue reservoir cell eradication poses a potential cost to these types of immunotherapy, especially in cases such as CNS reservoirs, where cells have limited regeneration capacity but make vital functional contributions to quality of life (186). Thus, alternative gene-editing approaches to specifically excise integrated viral DNA from infected cells (227) may be needed in combination with mAb-based approaches to achieve such a “cure.”

In an alternative definition, a “cure” may be functionally described as undetectable levels of virus in the absence of additional therapy. Such a “functional cure” may be more feasible by the Ab-based strategies described above, with particular emphasis on the life-long delivery of immunotherapy (gene or cellular therapy) or the stimulation of sufficiently broad and potent autologous immune responses for life-long immune surveillance.

## Conclusion

Preclinical studies of bNAbs to prevent and treat SHIV infection in macaques and Phase I human clinical trials demonstrating reduction of viral load and even reservoir size support the clinical utility and potential of bNAbs for prevention, postexposure prophylaxis, and therapy of acute and chronic infection. Observed and potential limitations of bNAbs noted thus far in these recent studies include the selection of resistant viral populations, immunogenicity resulting in the development of antidrug (Ab) responses, and the potentially toxic elimination of reservoir cells in regeneration-limited tissues. Opportunities to improve the utility of HIV Abs address these challenges and build upon each other as the timing/stage of infection progresses. Before exposure, bNAbs’ ability to prevent infection by neutralization may be improved by increasing serum half-life to necessitate less frequent administration, delivering genes for durable *in vivo* expression, and targeting bNAbs to sites of exposure. After exposure and/or in the setting of acute infection, bNAb use to prevent/reduce viral reservoir establishment and spread may be enhanced by increasing the potency with which autologous adaptive immune responses are stimulated, clearing acutely infected cells, and preventing cell–cell transmission of virus. In the setting of chronic infection, bNAbs may better mediate viral remission in combination with ARTs and/or LRAs, by targeting additional markers of tissue reservoirs or infected cell types, or by serving as targeting moieties in engineered cell therapy. Finally, various combinations of the described bNAb applications may play a role in the development of a true “cure” for HIV to eradicate HIV entirely, although the risk of eliminating certain reservoir tissue cells may encourage the use of alternative strategies to eliminate viral DNA from latent cells without eradicating the cells. In conclusion, bNAbs are potent and promising agents for HIV prevention and treatment at various stages of infection. Their sole use as therapy faces challenges of viral evasion, immunogenicity, and reservoir latency, which can be combatted by employing various, often complementary strategies in combination with each other and/or existing ART regimens. While the clinical use of HIV Abs has never been closer, remaining studies to precisely define, model, and understand the complex roles and dynamics of HIV Abs and viral evolution in the context of the human immune system and anatomical compartmentalization will be critical to optimizing their clinical safety and efficacy.

## Author Contributions

CH wrote and MA reviewed this article.

## Conflict of Interest Statement

The authors declare that the research was conducted in the absence of any commercial or financial relationships that could be construed as a potential conflict of interest.
